# Hemorrhagic Aspects of Gaucher Disease

**DOI:** 10.5041/RMMJ.10173

**Published:** 2014-10-29

**Authors:** Hanna Rosenbaum

**Affiliations:** 1 Department of Hematology and Bone Marrow Transplantation, Rambam Health Care Campus, Haifa, Israel and; 2 Bruce Rappaport Faculty of Medicine, Technion–Israel Institute of Technology, Haifa, Israel

**Keywords:** Enzyme replacement therapy, Gaucher disease, glucocerebroside, hemostatic abnormalities

## Abstract

Gaucher disease (GD) is an inherited lysosomal disorder, originating from deficient activity of the lysosomal enzyme glucocerebrosidase (GCase). Normally, GCase hydrolyzes glucocerebroside (GC) to glucose and ceramide; however, impaired activity of this enzyme leads to the accumulation of GC in macrophages, termed “Gaucher cells.” Gaucher disease is associated with hepatosplenomegaly, cytopenias, skeletal complications and in some forms involves the central nervous system.

Coagulation abnormalities are common among GD patients due to impaired production and chronic consumption of coagulation factors. Bleeding phenomena are variable (as are other symptoms of GD) and include mucosal and surgical hemorrhages.

Four main etiological factors account for the hemostatic defect in GD: thrombocytopenia, abnormal platelet function, reduced production of coagulation factors, and activation of fibrinolysis. Thrombocytopenia relates not only to hypersplenism and decreased megakaryopoiesis by the infiltrated bone marrow but also to immune thrombocytopenia. Autoimmunity, especially the induction of platelet antibody production, might cause persistent thrombocytopenia.

Enzyme replacement therapy reverses only part of the impaired coagulation system in Gaucher disease. Other therapeutic and supportive measures should be considered to prevent and/or treat bleeding in GD.

Gaucher patients should be evaluated routinely for coagulation abnormalities especially prior to surgery and dental and obstetric procedures.

## INTRODUCTION

Gaucher disease (GD) is an autosomal recessive inherited disorder of glycolipid storage, caused by a deficiency of the lysosomal enzyme glucocerebrosidase (GCase), which hydrolyzes glucocerebroside (GC) to glucose and ceramide, leading to the accumulation of GC in macrophages and resulting in multiorgan involvement.^1^ The macrophages of mononuclear origin with GC-laden lysosomes (named “Gaucher cells”) infiltrate the reticuloendothelial system and involve the liver, spleen, lung, and bone marrow, causing hepatosplenomegaly, anemia, thrombocytopenia, bone disease, and occasionally neurological involvement.^2^–^4^ Three GD types are characterized by the absence (type 1) or presence of neurologic involvement during childhood (type 2) or adolescence (type 3).^1^

Type 1 Gaucher disease (GD1) is panethnic but particularly common among the Ashkenazi Jewish population.^5^ Gaucher disease type 1 may present with a wide range of clinical manifestations, including various hemostatic abnormalities.

Enzyme replacement therapy (ERT) for GD1 has been available since 1991, first as human placenta-derived enzyme (alglucerase, Ceredase^®^, Genzyme, a Sanofi Company, Cambridge, MA, USA) and since 1994 as imiglucerase (Cerezyme^®^, Genzyme), a human recombinant form of the enzyme. A second recombinant human enzyme replacement therapy, velaglucerase-alfa (VPRIV) (Shire Human Genetic Therapies, Dublin, Ireland)^6^,^7^ was approved in 2010, and a third, taliglucerase-alfa (Elelyso™, Protalix, Carmiel, Israel) which is a plant cell-expressed acid β-glucocerebrosidase, was approved in the United States and other countries in 2012.^8^,^9^

Enzyme replacement therapy is beneficial for most hematological manifestations and some aspects of bone disease in GD patients.^10^,^11^ Enzyme replacement therapy reduces spleen and liver volumes, reverses or ameliorates the cytopenias following 12–24 months of treatment, and is considered the gold standard treatment for Gaucher type 1 and type 3 patients.^12^

While the hallmark of GD1 is organomegaly due to recurrent infarction, fibrosis, and extramedullary hematopoiesis, hemorrhagic phenomena are frequent, being the presenting symptom in 43% of Gaucher patients.^13^

The main hemorrhagic manifestations in GD patients are mucosal, including nose, gingiva, heavy menstrual bleeding, and hemorrhages induced by minor trauma, surgery, or labor.^14^,^15^ Spontaneous iliopsoas hematomas were reported in six GD patients as unusual severe bleeding events.^16^–^19^

Various coagulation defects contribute to hemorrhagic diathesis in GD, including thrombocytopenia, thrombocytopathy, clotting factor deficiencies, and enhanced fibrinolysis (Table 1).

**Table 1.** Hemostatic Abnormalities in Gaucher Disease: Evaluation and Therapeutic Approach.

	**Diagnostic Methods**	**Therapeutic Approach**
Thrombocytopenia	Complete blood count	Enzyme replacement therapy (ERT)
	Platelet < 100,000/mm^3^	Consider increasing enzyme dose
	Exclude “pseudo-thrombocytopenia” [Platelet aggregates]	Follow ITP guidelines in cases of immune thrombocytopenia
	Bone marrow aspiration and biopsy in patients with persistent thrombocytopenia	Consider IVIG, rituximab, and thrombopoietin receptor analogues
		Splenectomy only in life-threatening thrombocytopenia
Thrombocytopathy	Platelet aggregation tests with: adenosine-diphosphate, epinephrine, collagen, ristocetin	Evaluate prior to orthopedic surgery ERT
	Platelet adhesion tests	
Clotting factors	Prothrombin time	ERT
	Activated partial thromboplastin time	Coagulation factor concentrates for postoperative hemorrhage
	Mixing tests with normal plasma	
	Coagulation factor level	
	von Willebrand factor level	
Fibrinolysis	D-dimer	ERT to decrease coagulation activation
	Plasminogen	
	Alfa 2-antiplasmin	

IVIG, intravenous immunoglobulin.

## THROMBOCYTOPENIA

Bleeding complications in GD are frequently attributed to thrombocytopenia, which commonly occurs due to hypersplenism and/or bone marrow infiltration by Gaucher cells, compromising megakaryopoiesis. Stein et al. reported the presence of focal splenic lesions in GD patients, which was shown to be associated with greater degrees of thrombocytopenia, and lower response of thrombocytopenia to enzyme therapy.^20^

Gaucher disease appears to be associated with an increased incidence of immunological disorders, such as immune thrombocytopenia (ITP).^21^,^22^ The controversial issue of platelet-associated antibodies in the diagnosis of ITP in GD patients was previously presented in a case report and in an abstract form. Further studies are warranted to assess the role of platelet-associated antibodies in ITP observed in GD patients.

Hemorrhagic complications appear to be mainly related to the severity of thrombocytopenia. Others believe that platelet count alone may not be a reliable indicator of bleeding risk in GD patients.^23^ A higher risk of bleeding during (orthopedic) surgery is considered with a platelet count below 20×10^9^/L.^24^ Thrombocytopenia frequently normalizes within 1 to 2 years of ERT in patients with an intact spleen and moderate baseline thrombocytopenia.^25^ Persistent thrombocytopenia in GD patients treated with ERT for over 4 years relates to refractory splenomegaly. Therefore, life-threatening thrombocytopenia may be one of the few circumstances where splenectomy may still be justified in GD.^17^,^26^ The use of rituximab, high-dose gammaglobulin, and thrombopoietin receptor analogues should be considered as therapeutic modalities in Gaucher patients with immune thrombocytopenia.

## THROMBOCYTOPATHY

Hemorrhagic diathesis has been noted in GD patients with platelet counts of more than 100×10^9^/L and normal clotting studies, suggesting platelet function abnormalities.^27^ Platelet functions, namely adhesion and aggregation, were studied in cohorts of GD1 patients, resulting in detection of relatively common function abnormalities in different GD1 patient populations. Mucosal bleeding was reported in GD patients in association with abnormal platelet adhesion; however, the mechanism of reduced platelet adhesion in patients with type GD1 is not clear.^16^ Increased plasma levels of glucocerebroside in patients with GD may affect platelet activation.^28^,^29^

Reduced platelet aggregation in response to all agonists was observed with a relatively high incidence of unexplained bleeding tendency in these GD1 patients.^23^,^30^,^31^ Interestingly, platelet counts and platelet aggregation were shown to improve following ERT.^17^,^30^,^31^

## CLOTTING FACTOR DEFICIENCIES

Reduced activity of coagulation factors, namely factors II (FII), V (FV), VII (FVII), IX (FIX), and X (FX), was reported in GD.^17^,^30^,^32^ A high frequency of FXI deficiency was detected among Ashkenazi patients with type 1 GD. This may be explained by a relatively common concurrence of both genetic disorders in this population.^18^,^33^ Hollak et al. reported that the degree of clotting factor deficiencies was highly variable, with coagulation factor levels as low as 15% in some GD patients. The most frequently encountered clotting factor abnormalities were deficiencies of FV, FX, and FII.^17^ The lowest levels were observed in FII, FVII, and FX. Severe deficiencies of FII, FVII, and FX were usually present in the same patients.^34^,^35^ Factor V activity was only mildly reduced in some of the patients. Plasma levels of thrombin–antithrombin complexes were found to be elevated in 46% of the patients, suggesting that the deficiencies in clotting factors were due to consumption caused by enhanced ongoing low-grade activation of coagulation (especially in patients with splenomegaly) rather than due to impaired synthesis and could be partly restored by ERT.^17^,^27^

Giona et al. reported that all coagulation factors which were low before ERT returned to normal values after a median time of 28 months (range 6–63) since the start of ERT.^30^ Hollak et al.^17^ suggested that the effect of ERT on coagulation parameters might be related to the reduction of spleen volume.

## ENHANCED FIBRINOLYSIS

It was also suggested that splenic intravascular coagulation and fibrinolysis might have a role in GD, especially in patients with splenomegaly. Gerrits et al.^35^ postulated that splenomegaly is associated with chronic consumption coagulopathy. Mitrovic et al.^18^ found correlation between Gaucher cell burden measured by chitotriosidase values and D-dimer, suggesting the relationship between pro-inflammatory cytokines, coagulation activation, fibrinolysis, and natural anticoagulant consumption without overt disseminated intravascular coagulation (DIC).

However, Hollak et al.^17^ reported reduced levels of plasminogen and a2-antiplasmin in 37% and 20% of the studied GD1 patients, respectively. The overall fibrinolytic activity, reflected by plasma levels of plasmin–a2-antiplasmin complexes and D-dimer, was enhanced, indicating that the depletion of fibrinolytic proteins is the result of continuing activation of the fibrinolytic system.

## CONCLUSION

Hemorrhagic diathesis is common in Gaucher patients and is caused by various abnormalities of the coagulation pathways, including platelet number and function, coagulation factors, and fibrinolysis. The visceromegaly caused by Gaucher cell infiltration contributes to abnormal production activation and consumption of coagulation factors. Enzyme replacement therapy corrects only part of the coagulopathies in Gaucher disease.

## Abbreviations:

DIC: disseminated intravascular coagulation;
ERT: enzyme replacement therapy;
FII: factor II;
FIX: factor IX;
FV: factor V;
FVII: factor VII;
FX: factor X;
FXI: factor XI;
GC: glucocerebroside;
GD: Gaucher disease;
GD1: type 1 Gaucher disease;
ITP: immune thrombocytopenia.
